# Supplementary material for: Comparison of the Ammonia Trapping Performance of Different Gas-Permeable Tubular Membrane System Configurations
Source: Membranes (Basel). 2022 Nov 5;12(11):1104. doi: 10.3390/membranes12111104 (PMC9699080; doi:10.3390/membranes12111104)
Supplement: Supplementary file 1 [file membranes-12-01104-s001.zip › membranes-1977338-supplementary.pdf]

Comparison of the Ammonia Trapping Performance of Different Gas-Permeable Tubular Membrane System Configurations

M. Soto-Herranz, M. Sánchez-Báscones, M.C. García-González, P. Martín-Ramos

SUPPLEMENTARY MATERIAL

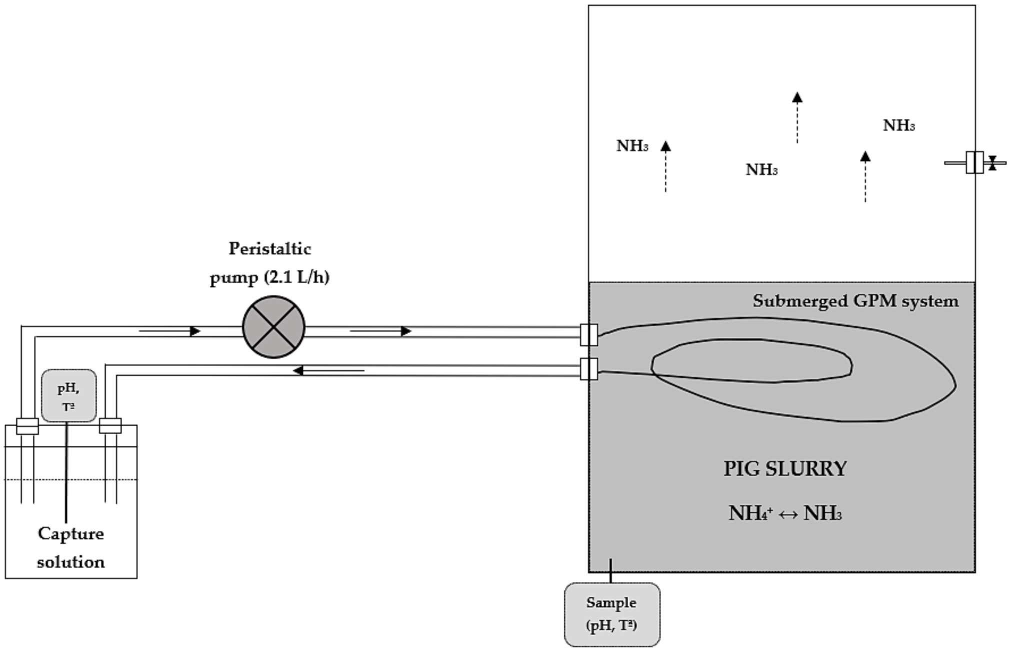

Figure S1. Scheme of the NH_3 capture process in the GPM S1 system, consisting of a submerged membrane without agitation or aeration of pig slurry.

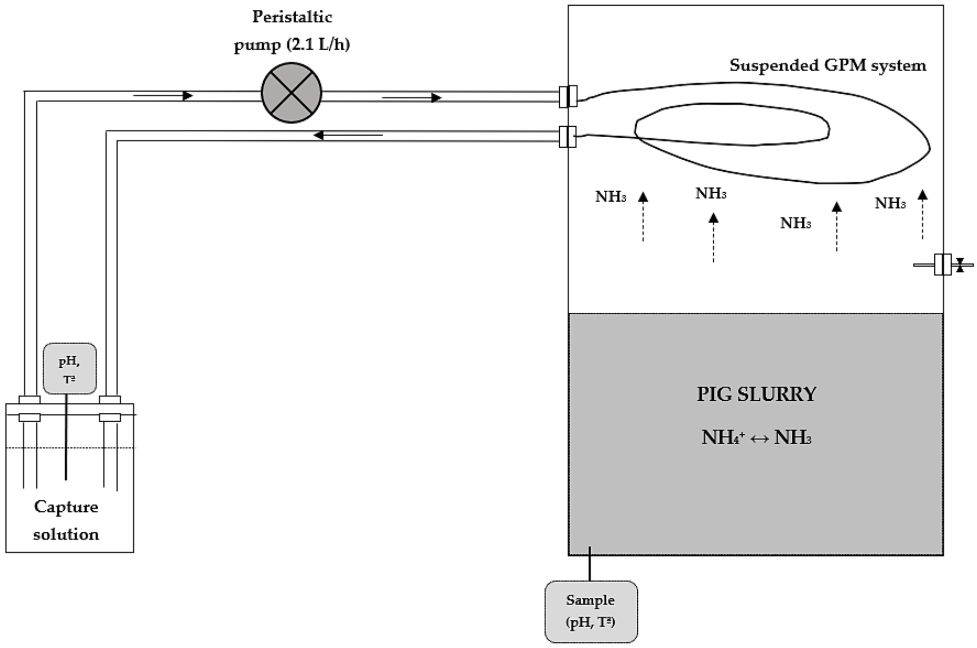

Figure S2. Scheme of the NH_3 capture process in the GPM S2 system, consisting of a suspended membrane without agitation or aeration of pig slurry.

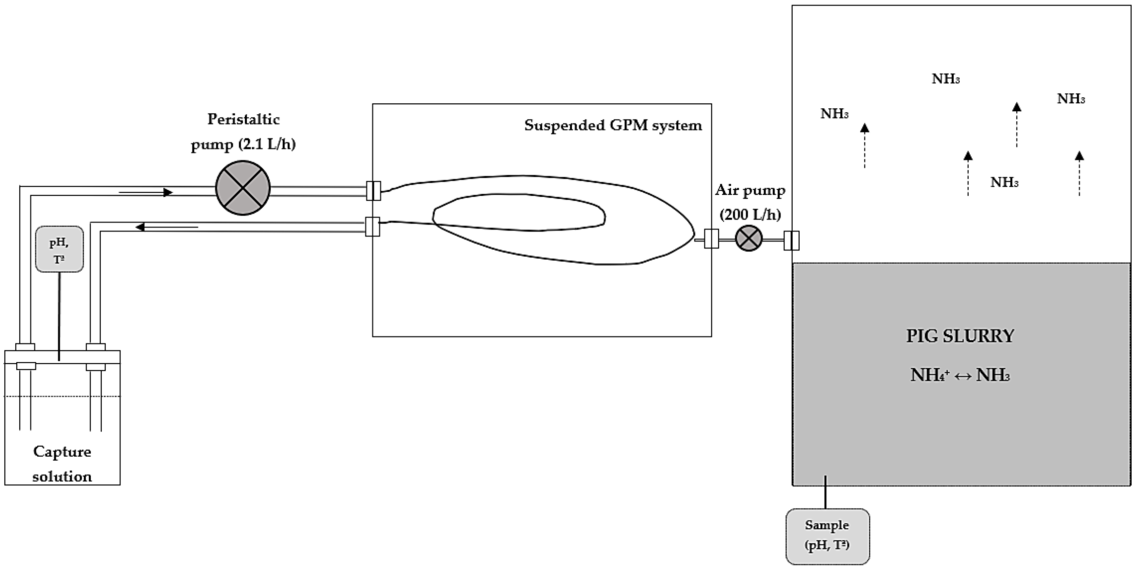

Figure S3. Scheme of the NH_3 capture process in the GPM S3 system, consisting of a membrane suspended in a compartment attached to the slurry treatment chamber, from which the NH_3-laden air is sucked in.

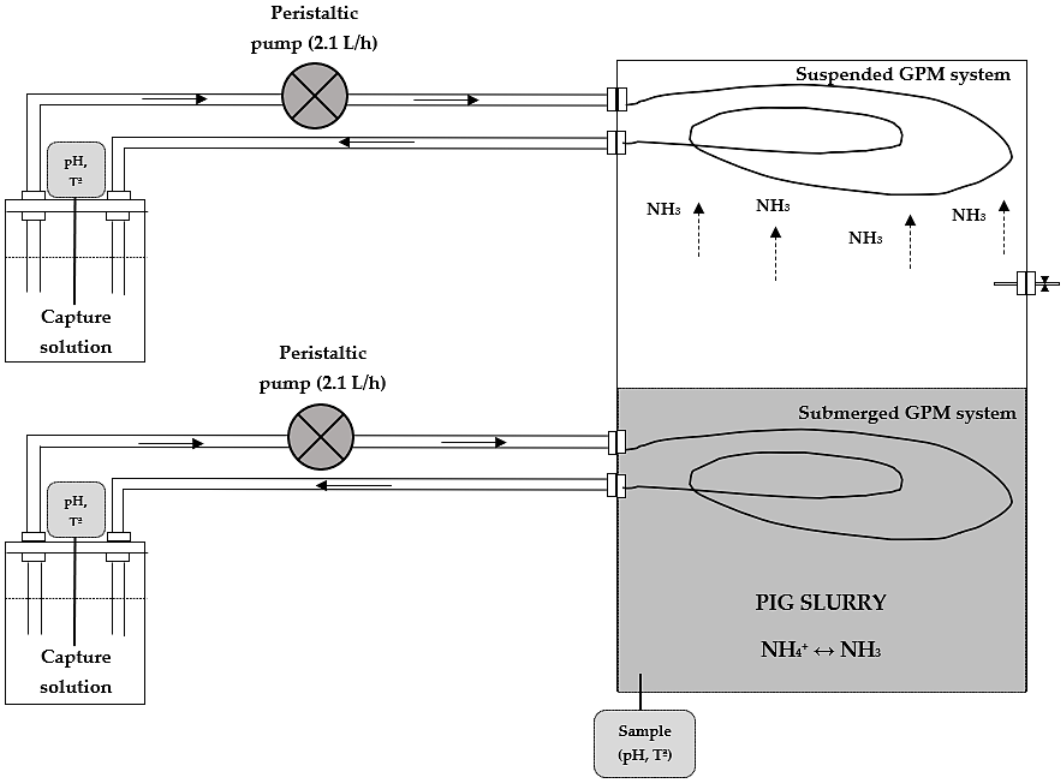

Figure S4. Scheme of the NH_3 capture process in the GPM S4 system consisting of a submerged and a suspended membrane without agitation and aeration of pig slurry.

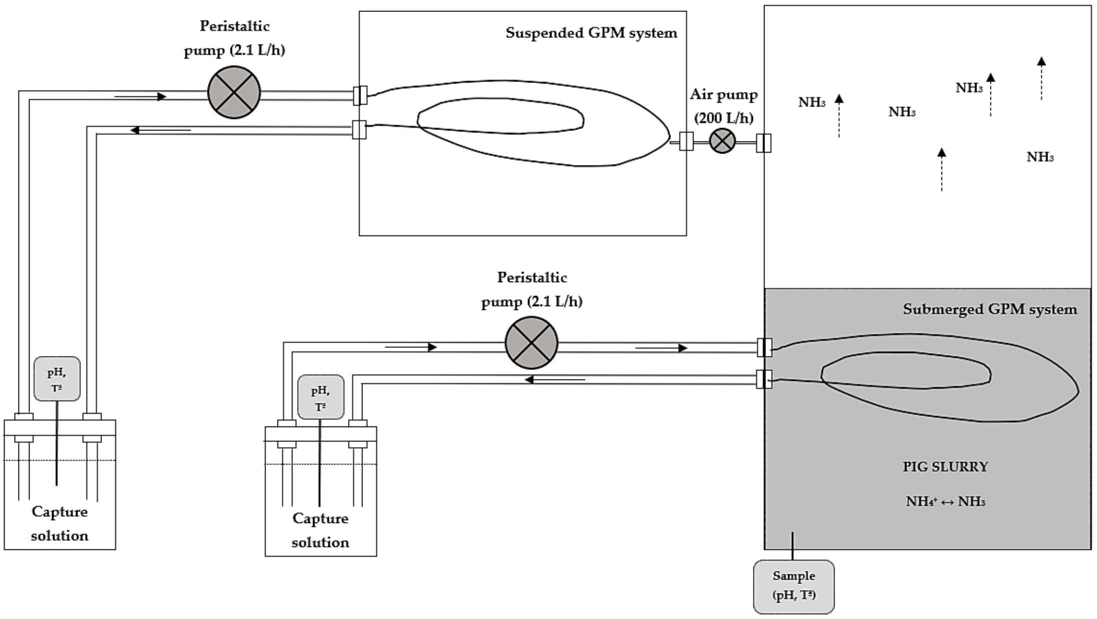

Figure S5. Scheme of the NH_3 capture process in the GPM S5 system, consisting of a suspended membrane in a compartment attached to the slurry treatment chamber, from which the NH_3-laden air is sucked in, and a submerged membrane without agitation and aeration of pig slurry.

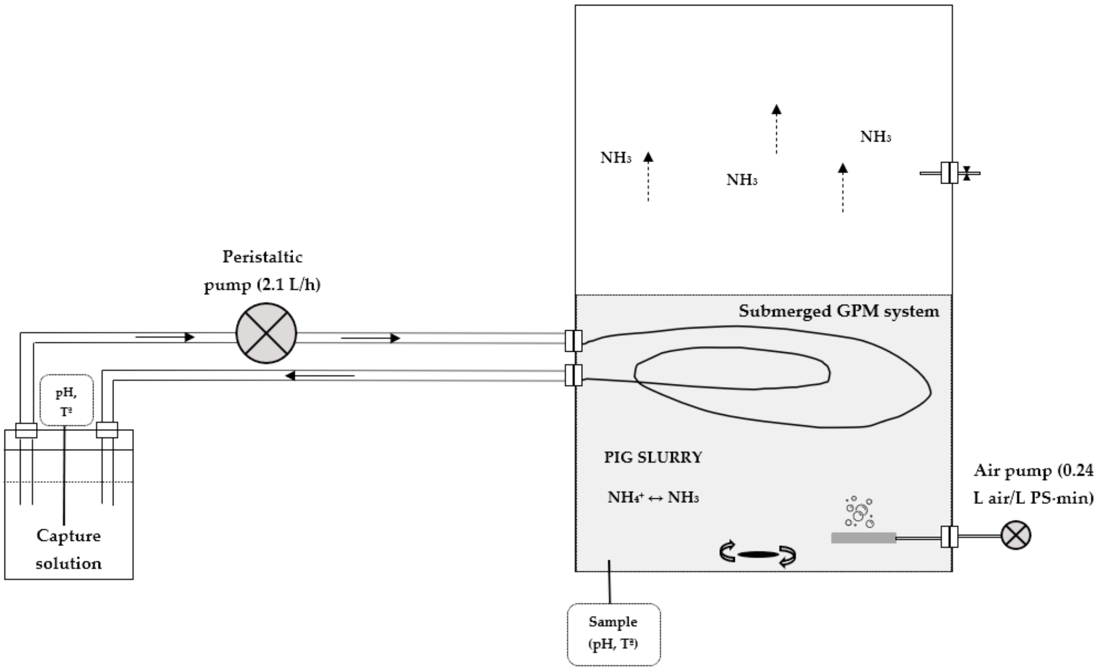

Figure S6. Scheme of the NH_3 capture process in the GPM S6 system, consisting of a submerged membrane with agitation and aeration of pig slurry.

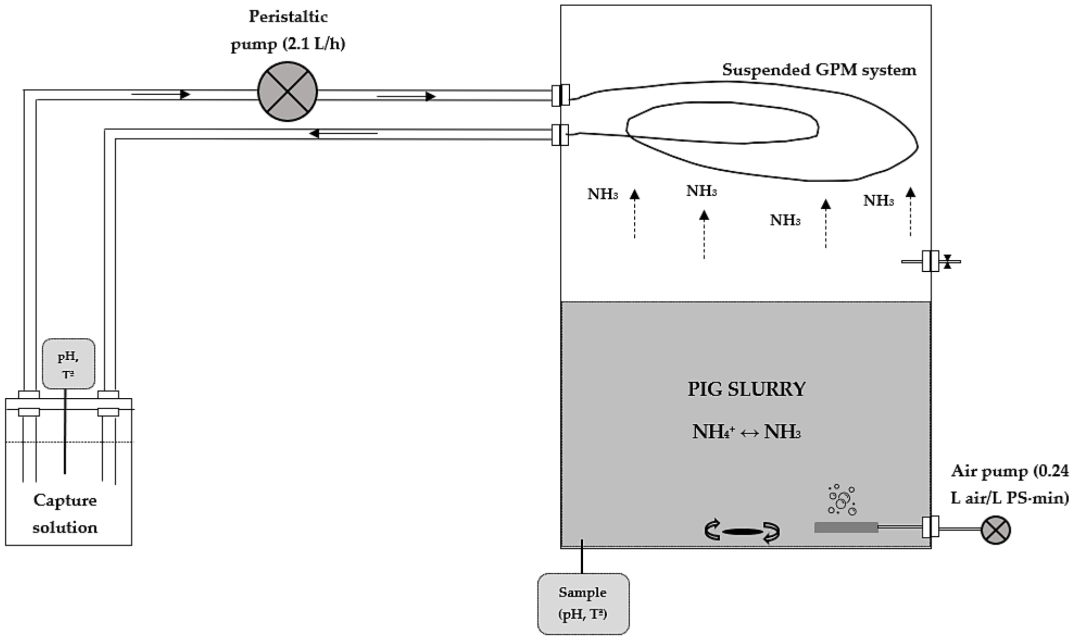

Figure S7. Scheme of the NH_3 capture process in the GPM S7 system, consisting of a suspended membrane with agitation and aeration of pig slurry.

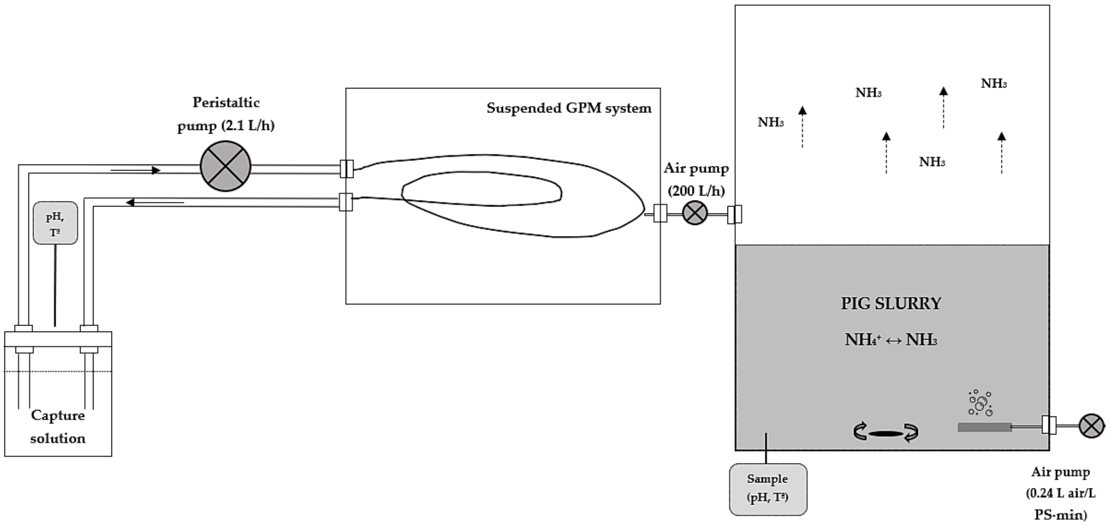

Figure S8. Scheme of the NH_3 capture process in the GPM S8 system, consisting of a membrane suspended in a compartment attached to the slurry treatment chamber with agitation and aeration of pig slurry, from which NH_3-laden air is sucked in.

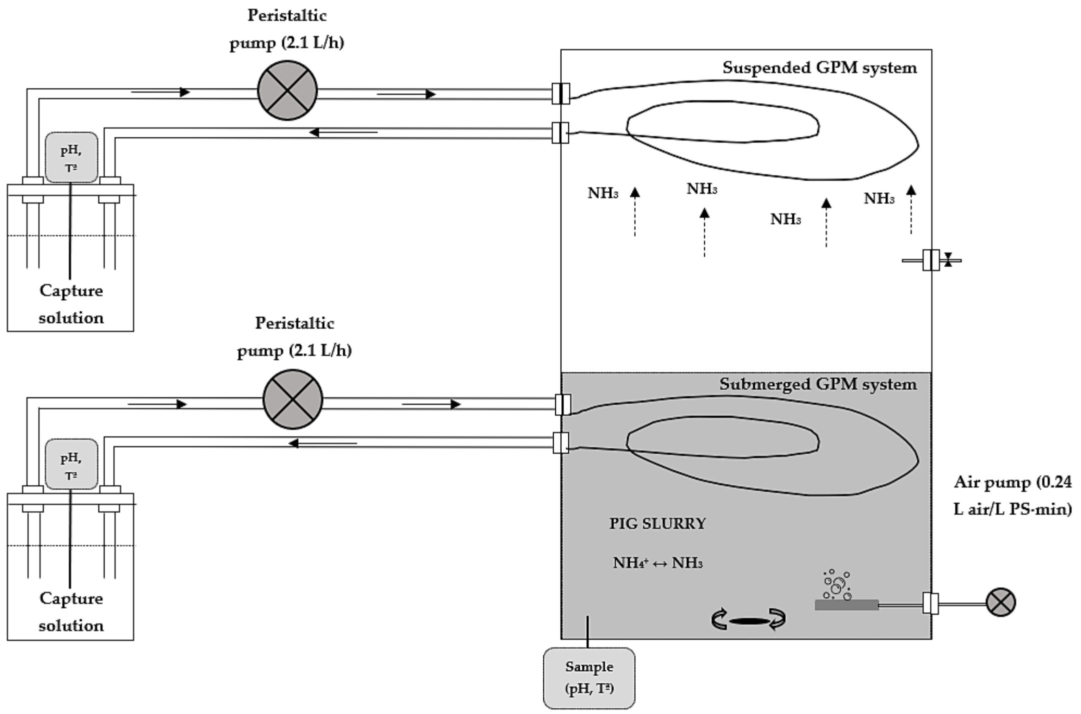

Figure S9. Scheme of the NH_3 capture process in the GPM S9 system, consisting of a submerged and a suspended membrane with agitation and aeration of pig slurry.

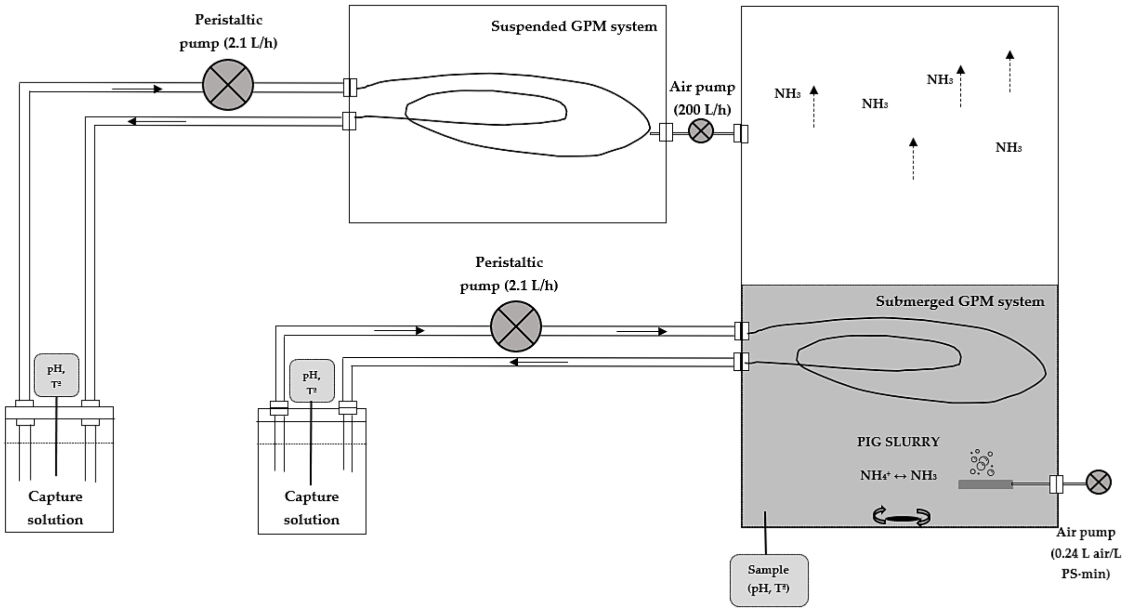

Figure S10. Scheme of the NH_3 capture process in the GPM S10 system, consisting of a suspended membrane in a compartment attached to the slurry treatment chamber, from which the NH_3-laden air is sucked in, and a submerged membrane with agitation and aeration of pig slurry.

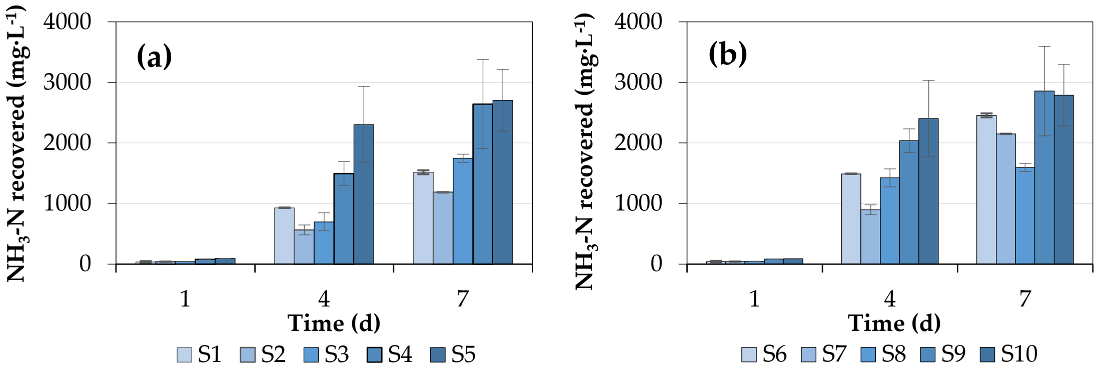

Figure S11. Evolution of NH_3-N concentration in the acid capture solution in (**a**) systems S1-S5 without slurry aeration and agitation; and (**b**) systems S6-S10 with slurry agitation and aeration. The meaning of the abbreviations is presented in section 2.2.
